# Health-seeking amid conflict and climate stress: how climate disaster shapes maternal and child healthcare behavior in fragile and conflict-affected settings

**DOI:** 10.1186/s12913-026-14744-3

**Published:** 2026-05-20

**Authors:** Gbadebo Collins Adeyanju, Liliana Abreu, Pia Schrage, Johanna Brinkel, Rabiu I. Jalo, Musa Muhammad Bello, Aminatu A. Kwaku, Aisha Aliyu Abulfathi, Muhammad Ibrahim Jalo, Ahmad Mahmud, Max Schaub

**Affiliations:** 1 Media and Communication Science, University of Erfurt, Erfurt, Germany; 2 Centre for Empirical Research in Economics and Behavioral Science (CEREB), University of Erfurt, Erfurt, Germany; 3 Psychology and Infectious Diseases Lab (PIDI), University of Erfurt, Erfurt, Germany; 4 Adjunct Professor of Global Health, RHIBMS University, Buea, Cameroun; 5 Department of Politics and Public Administration, University of Konstanz, Konstanz, Germany; 6 Willy Brandt School of Public Policy, University of Erfurt, Erfurt, Germany; 7 Department of Infectious Disease Epidemiology, Bernhard Nocht Institute for Tropical Medicine (BNITM), Hamburg, Germany; 8 Department of Community Medicine, Faculty of Clinical Sciences, Bayero University Kano, Kano, Kano State Nigeria; 9 Department of Community Medicine, Aminu Kano Teaching Hospital, Kano, Kano State Nigeria; 10 University of Maiduguri Teaching Hospital and University of Maiduguri, Maiduguri, Borno State Nigeria; 11 Women and Children Hospital, Damaturu, Yobe State Nigeria; 12 Modibbo Adama University Teaching Hospital and Modibbo Adama University, Yola, Adamawa State Nigeria; 13 Faculty of Business, Economics and Social Sciences, University of Hamburg & WZB Berlin Social Science Center, Berlin, Germany

**Keywords:** Floods, Climate disasters, Maternal healthcare services, Antenatal care, Immunization, Child healthcare services, Women and children, Nigeria, Sub-Saharan Africa, Climate change

## Abstract

**Background:**

Floods can have significant adverse health consequences, placing an additional burden on healthcare systems and altering healthcare-seeking behavior. However, its effects on the health-wellbeing of vulnerable groups, such as pregnant women and children, in resource-constrained and conflict-affected Nigerian communities remain underexplored. This study examines the impact of the 2024 floods in Borno State, Nigeria, on maternal and child healthcare-seeking behavior.

**Methods:**

A qualitative phenomenological approach was employed, with semi-structured Focus Group Discussions serving as the primary data collection method. Participants were selected purposively, focusing on households with pregnant women and children under five years old. Six FGDs, each comprising eight participants, were conducted across six geopolitical constituencies. The data were transcribed, framed, coded and analyzed thematically using Nvivo.

**Results:**

The 2024 flood in Borno exacerbated existing vulnerabilities in social and healthcare infrastructure, disrupting essential maternal and child healthcare services. Public healthcare facilities were overwhelmed, with some healthcare facilities being repurposed as shelters, which further compounded the hardships experienced by vulnerable populations. The flood led to massive displacement, emotional trauma and increased health risks, particularly among children and pregnant women. The flood led to outbreaks of infectious diseases, long-term deterioration in access to antenatal care and immunization services, destruction of healthcare facilities, loss of medical supplies, and damage and contamination of existing drugs, as well as the destruction of cold chain systems critical for vaccine preservation. Cultural beliefs and environmental mismanagement further intensified the crisis.

**Conclusion:**

The findings underscore the urgent need for climate-resilient healthcare systems to improve disaster preparedness and management in the context of climate change. The resulting displacement, emotional trauma and health risks, particularly for marginalized groups, emphasize the need for integrated, climate-resilient systems that safeguard both infrastructure and vital public healthcare services.

**Supplementary Information:**

The online version contains supplementary material available at 10.1186/s12913-026-14744-3.

## Introduction

Floods are among the most frequent and destructive natural disasters globally, with a significant impact on the environment, economy, and human health [1, 2]. Over the past four decades, the frequency of flood-related events and associated mortality has increased, with an estimated 1.8 billion people, I.e., roughly one-fifth of the global population are exposed to frequent flooding [3]. Climate change is expected to exacerbate these trends, producing more severe, prolonged and frequent floods [2, 4].

Floods affect human health both directly and indirectly. Direct effects include injury, drowning, and trauma from collapsing infrastructure, while indirect effects include water supplies contamination, infectious and vector-borne diseases outbreaks, impaired access to healthcare services, and psychological stress [5, 6]. Vulnerable populations, particularly pregnant women, children, and the elderly, experience disproportionately more adverse outcomes, including maternal stress, impaired immune function, adverse birth outcomes, and long-term mental health sequelae in children [7–11].

During the peak of the rainy season in August 2024, Nigeria experienced severe flooding, affecting approximately 1.2 million people across 31 states and causing widespread displacement, infrastructure damage, and loss of agricultural land [12]. Maiduguri, the capital of Borno State, was one of the areas worst affected. Heavy rainfall caused the Alau Dam collapse, submerging nearly 70% of the city and affecting over 230,000 internally displaced persons (IDPs), including at least 14 healthcare facilities [12, 13]. The flooding damaged essential infrastructure, disrupted access to healthcare services and increased the risk of infectious disease outbreaks, including cholera, measles and enteric fever [13].

Flooding also affected healthcare utilization and can lead to a range of health issues among residents exposed to floodwaters, including waterborne diseases, respiratory problems, skin conditions and psychological disorders such as stress and post-traumatic effects [14–18]. Studies have documented increased maternal and child morbidity and mortality following floods, including miscarriage and stillbirth, as well as decreased uptake of antenatal care (ANC) and immunization services [1, 7, 8, 19]. Flood-related displacement and damage to healthcare facilities, including disruption to the cold chain for vaccines, further reduces access to essential services. Evidence from other settings, such as South Korea and the United States, indicates that floods can alter healthcare-seeking behavior, resulting in increased visits for injuries and infectious diseases, as well as reduced utilization of maternal healthcare services [20, 21].

Despite this evidence, there is limited research on the impact of flooding on the maternal and child healthcare-seeking behavior in resource-constrained and conflict-affected settings, such as Borno State. Empirical studies often focus on direct health outcomes rather than broader healthcare services utilization or behavioral responses [22, 23]. Furthermore, there is a paucity of empirical evidence examining healthcare-seeking behavior in the context of climate-related disasters in conflict-affected Nigerian communities.

This study therefore aims to explore how a climate event such as the flooding in Maiduguri in September 2024 affected healthcare-seeking behavior for maternal and child health, as well as how knowledge of climate change influences this behavior. By focusing on a setting that is both resource-constrained and conflict-affected, this research provides empirical evidence on the intersection of climate disasters, vulnerability and health system resilience.

## Methods

### Study design

A qualitative phenomenological approach was employed to explore the lived experience of residents regarding healthcare-seeking for maternal and child healthcare following the flooding. Focus Group Discussions (FGDs) were used as the primary methods of data collection to enable an in-depth exploration of participants´ perceptions, challenges and coping strategies. The study uses FGD to leverage group interaction, producing richer and more rounded insights than individual interviews. Moreover, by facilitating dialogue among individuals with shared experiences, FGDs cultivate a microcosm of a thinking society that enables the exploration of collective meanings, sentiments, and convictions.

### Study setting

The study was conducted in Maiduguri, the capital of Borno State in Nigeria, which was the region most severely affected by the flooding in September 2024. As of 2022, Maiduguri’s population was estimated to be around two million [24]. The study focused on the two local government areas (LGAs) within Maiduguri: I.e., Maiduguri and Jere LGAs. These LGAs comprise 27 political wards (15 in Maiduguri and 12 in Jere). Within each LGA, the three most severely affected wards were selected as clusters, resulting in a total of six clusters for the study.

### Study sampling and participants’ characteristics

Participants were recruited using purposive sampling to identify households with at least one pregnant woman and/or at least one child under five years of age. Each FGD comprised eight participants to enable rich, in-depth interaction. Gender balance was maintained through equal representation of male and female participants. In total, six FGDs were conducted across six selected clusters, with three FGDs in each local government area (LGA), yielding a total sample of 48 participants. Participants were primarily household heads drawn from flood-affected communities, with half recruited from households with at least one pregnant woman and the remaining half from households with at least one child under five years old.

Recruitment was undertaken in flood-affected communities in Borno State to ensure inclusion of caregivers with direct experience of pregnancy and childcare under crisis conditions. Community leaders and trusted local gatekeepers facilitated access to households and supported recruitment by identifying eligible participants and extending invitations on behalf of the research team. Participants included women and men of reproductive age engaged in a range of occupations and household arrangements, reflecting the socioeconomic diversity of the study settings. Prior to data collection, the study aims, procedures, potential risks, and benefits were clearly explained. Written informed consent was obtained on-site before the start of each FGD, and participants were encouraged to ask questions. Confidentiality and anonymity were explicitly addressed, including the use of anonymized identifiers in transcripts and reports, and consent for audio recording was obtained from all participants before discussions commenced.

### Inclusion criteria

- Residents of flood-affected communities in Maiduguri or Jere LGAs during the 2024 flooding.
- Individuals from households with at least one pregnant woman and/or one child under five years old.
- Adult participants (men or women of reproductive age), particularly household heads or primary caregivers.
- Individuals willing and able to provide informed consent and participate in focus group discussions.
- Participants with direct experience of healthcare-seeking for maternal and/or child healthcare during the flood.

### Exclusion criteria

- Individuals not residing in the selected flood-affected communities during the 2024 flooding.
- Households without pregnant women or children under five years old.
- Individuals below 18 years of age or unable to provide informed consent.
- Persons not directly involved in caregiving or healthcare decision-making within the household.
- Individuals unable to participate effectively in FGDs due to severe illness, cognitive impairment or communication barriers.

### Data collection and analysis

All data were collected between February 22 - March 10, 2025 (five months post-flood) using a semi-structured interview guide (see supplementary file). Each interview lasted an average of 90 minutes. They were audio-recorded and transcribed verbatim. The transcripts were organized in a framed spreadsheet and analyzed thematically using NVivo software. Thematic analysis was conducted in accordance with Braun and Clarke’s protocol [25], which involves identifying patterns and themes in the data. This involved guide comprising the following steps: familiarization with the data; generation of initial codes; development of broad themes; review and refinement of themes; conceptualization of themes; and writing of findings [26]. A hybrid coding approach was employed, combining deductive codes based on the study objectives with inductive codes that emerged from the data. Participants’ data were anonymized and coded based on their LGAs (Maiduguri, coded as MMC, or Jere), FGD number (e.g., 1, 2, 3, etc.) and a personal identifier (e.g., A, B, C, etc.), as shown in Table 1. Participants were identified as MMC_1A, MMC_2D, Jere_1C, Jere_2F, and so on. Data saturation was reached by the fifth FGD, as iterative analysis showed that no new themes or codes were emerging and participants’ responses had become repetitive, consistently reinforcing previously identified patterns.

### Reflexivity statement

This qualitative study was conducted in a post-disaster, conflict-affected setting among vulnerable populations, necessitating heightened reflexivity and ethical consideration. FGD moderators were selected based on their academic qualifications and experience in humanitarian contexts. They received training in qualitative interviewing, trauma-informed approaches and non-leading questioning techniques. They were fluent in local languages and culturally competent, facilitating trust and rapport. Strict safety and ethical protocols were applied throughout, including informed consent, confidentiality, and context-specific risk assessments. Regular debriefings and supervisory support ensured ongoing reflexivity, researcher wellbeing, and adherence to ethical standards during data collection and analysis.

## Results

Of the 48 participants in the six FGDs, 24 were women and 24 were men. Participants reported having between one and 12 children, totaling 248 children across all participants. The dominant occupations among women were fishing net making and cap knitting, while men were predominantly artisans and traders/businessmen. Participants ranged in age from 20 to 60 years old, with an average age of 36. Table 1 summarizes the key characteristics of the study participants.

**Table 1:** Characteristics of participants

Sn	Participant ID	FGD ID	Gender		Age	No of Children	Occupation
1	A	MMC_1	Female	24		3	Cap Knitting
2	B	MMC_1	Female	40		4	Cap Knitting
3	C	MMC_1	Female	30		3	Fashion Design
4	D	MMC_1	Female	36		6	Tailoring
5	E	MMC_1	Female	25		4	Cap Knitting
6	F	MMC_1	Female	20		2	Cap Knitting
7	G	MMC_1	Female	40		7	Sells Charcoal
8	H	MMC_1	Female	20		2	Cap Knitting
9	A	MMC_2	Male	35		1	Cap Knitting
10	B	MMC_2	Male	45		5	Teacher
11	C	MMC_2	Male	32		1	Bricklayer
12	D	MMC_2	Male	42		5	Teacher
13	E	MMC_2	Male	35		5	Medicine Vendor
14	F	MMC_2	Male	45		8	Businessman
15	G	MMC_2	Male	46		6	Trader
16	H	MMC_2	Male	33		3	Barber
17	A	MMC_3	Male	39		3	Businessman
18	B	MMC_3	Male	55		9	Mechanic
19	C	MMC_3	Male	39		5	Businessman
20	D	MMC_3	Male	38		6	Trader
21	E	MMC_3	Male	39		6	Cap Knitting
22	F	MMC_3	Male	55		5	Trader
23	G	MMC_3	Male	45		7	Trader
24	H	MMC_3	Male	58		7	Businessman
25	A	Jere_01	Female	33		7	Trader
26	B	Jere_01	Female	40		9	Cap Knitting
27	C	Jere_01	Female	30		4	Cap Knitting
28	D	Jere_01	Female	30		3	Trader
29	E	Jere_01	Female	32		6	Tailoring
30	F	Jere_01	Female	35		8	Trader
31	G	Jere_01	Female	33		12	Cap Knitting
32	H	Jere_01	Female	37		6	Cap Knitting
33	A	Jere_02	Female	30		3	Fishing Net Making
34	B	Jere_02	Female	33		6	Fishing Net Making
35	C	Jere_02	Female	35		7	Fishing Net Making
36	D	Jere_02	Female	30		5	Fishing Net Making
37	E	Jere_02	Female	35		6	Cap Knitting
38	F	Jere_02	Female	23		2	Cap Knitting
39	G	Jere_02	Female	25		3	Cap Knitting
40	H	Jere_02	Female	30		6	Tailoring
41	A	Jere_03	Male	30		6	Teacher
42	B	Jere_03	Male	25		2	Cap Washing
43	C	Jere_03	Male	30		5	Trader
44	D	Jere_03	Male	38		3	Farmer
45	E	Jere_03	Male	60		6	Trader
46	F	Jere_03	Male	51		5	Businessman
47	G	Jere_03	Male	45		7	Businessman
48	H	Jere_03	Male	43		8	Businessman

### Collapse of essential systems and healthcare services

#### Disruption of healthcare access and damage to infrastructure

Flooding caused a severe breakdown on essential public and healthcare services across the study areas. Hospitals, schools, and other facilities were either destroyed or repurposed as shelters for displaced people, leaving residents without access to routine medical care. Even facilities that remained structurally intact were often inaccessible or overwhelmed by displaced populations. Participants described the collapse of the local healthcare system as sudden and absolute:

When it happened, there was no hospital, chemist, or pharmacy where you can seek medical attention; the hospital near us was also affected” (Jere_1C). “When the floodwaters came, they went over the gate and completely inundated the hospital (…) flooding the patient wards and the pharmacy; the volume of water was simply too much (Jere_3G).

#### Loss and contamination of medical supplies and records

Floodwater also destroyed and contaminated medicines, healthcare records, and diagnostic materials. Participants reported that exposure to floodwater rendered drugs ineffective and significantly compromised healthcare delivery:

We have a nearby hospital, but unfortunately, the drugs have been contaminated by floodwater and had lost their potencies and or efficacies” (Jere_1D). “The shops including hospitals and chemists were all closed. The water damaged all the government-owned facilities (Jere_3A).

The destruction of medical documentation, including ANC and child vaccination cards, created additional barriers to access and care. Participants described being denied medical treatment because they were unable to provide proof of their previous medical history.

The flood damaged my child’s vaccination card, and when I went there, they said they would not give injection without the card. That is where I stopped the vaccination” (Jere_1F). “Unfortunately, I lost foodstuffs, clothes, and my ANC card, which has now made it impossible for me to attend ANC appointments, even as I approach my childbirth due date (MMC_3A).

These results highlight the vulnerability of healthcare systems in flood-affected areas. The loss of supplies and destruction of healthcare records all undermined the recovery process. The inability to resume essential maternal and child healthcare services contributed to long-term gaps in care and a decline in community trust in public healthcare institutions.

#### Healthcare workforce and service disruptions

The flooding brought the entire healthcare system to its knees, even after the floodwaters had receded. Facilities were understaffed, lacked essential medicines, and remained physically inaccessible due to damaged roads and infrastructure. This left communities unable to access even basic medical care for an extended period after the flood.

There were no doctors, no staff, and the medical records and other instruments were all destroyed by the flood” (Jere_1B). “There were no medical personnel available when we were finally able to access the hospital, and we found that some of the files had been destroyed by the flood, and even some of the drugs had been damaged” (Jere_1A). “Even when people manage to reach a hospital, medications are often unavailable” (MMC_2F).

### Household-level impact and healthcare burdens

#### Destruction of homes, property and livelihoods

Most participants reported extensive losses of homes, household assets, sanitation facilities, and livelihoods, resulting in displacement and heightened economic hardship:

The flooding has completely destroyed my house. I have lost everything, and I have children to care for. (…)” (MMC_2D). “It destroyed our homes, and we were disoriented. We faced serious challenges, losing our homes, food, clothing, and all our possessions (Jere_3F).

The loss of income-generating activities further constrained households’ ability to seek healthcare or purchase medicines, exacerbating existing socioeconomic vulnerabilities.

### Food and water insecurity

Flooding significantly disrupted access to safe food and clean drinking water. Participants reported reliance on contaminated water sources and spoiled food, leading to widespread illness:

The flood seriously affected our health and healthcare system in general… we were forced to ingest dirty water… some of us experienced stooling and vomiting, while others developed serious fevers” (MMC_3H). “When we returned, we had to throw away the food items we left behind because we could not eat them. My children had diarrhea because of eating the contaminated food we left behind. (Jere_3E).

A lack of safe water and food was reported as a major contributor to dehydration and illness, particularly among children, pregnant women and the elderly. These challenges persisted well beyond the immediate flooding period.

### Immediate and long-term physical and psychosocial health impacts

Most participants and their families experienced significant health problems during and after the flooding. Commonly reported conditions included fever, diarrhea, cholera, malaria, typhoid, skin rashes, and scabies, which participants attributed to exposure to contaminated water, poor sanitation, unhygienic conditions, and disrupted healthcare access:

Honestly, the floods have caused many diseases, such as fever and stomach pains. We later realized that this was caused by drinking the contaminated water… nearly all the children in the whole area contracted fever.” (MMC_1G). “During the flood, many people experienced itchy skin… After the flood, fever, diarrhea, stooling, and vomiting were rampant.” (MMC_3C). “Actually, the most severe diseases we faced were typhoid fever and diarrhea due to contamination of food and water (Jere_1A).

In addition to physical illness, participants reported sustained stress, anxiety, exhaustion, and emotional distress, reflecting the long-term psychosocial toll of the disaster.

### Disproportionate effects on vulnerable populations

#### Maternal healthcare challenges and unsafe deliveries

Pregnant women experienced profound barriers to accessing ANC, skilled birth attendance (SBA), and safe delivery environments during and after the floods:

The challenges pregnant women face includes a lack of suitable shelter… pregnant women were exposed to mosquitoes, hunger, anxiety, and more.” (MMC_2F). “Because the primary healthcare facility was flooded, most pregnant women gave birth at home, assisted by other women (Jere_3D).

Some women were forced to give birth in precarious environments and highly unsafe conditions, such as uncompleted buildings, makeshift shelters, or at roadsides, including while fleeing floodwaters. In some cases, they gave birth alone when they went into labor. The absence of SBA and the lack of access to functioning healthcare facilities created life-threatening situations for both mothers, and also newborns.

The flood seriously affected me… I suffered through it and even delivered my baby during that time. Afterward, my four other children and I became ill with symptoms like diarrhea and vomiting.” (MMC_3H). “During the floods, people narrowly escaped danger. We witnessed women in labor giving birth while on the run. We suffered a lot. (Jere_2G).

Participants also reported a significant increase in miscarriages and stillbirths following the flood. Pregnancy complications and losses were exacerbated by the physical and emotional stress caused by displacement, hunger, illness and fear.

When we returned, we found our house had collapsed, which terrified me, especially because my wife was pregnant. She fell ill several times and ultimately had a stillbirth” (Jere_3G). “My wife, who had just given birth when the flooding occurred, also couldn’t stand and lost the baby… she cannot use her legs now” (MMC_1H). “… pregnant women faced devastating outcomes, including pregnancy loss and stillbirths (MMC_3D).

#### Child health risks and malnutrition

Children faced heightened exposure to disease and malnutrition, the difficulty of keeping them safe during the disaster, as well as the challenges of obtaining medical care afterwards.

While many elderly individuals also experienced elevated blood pressure, tragically, children suffered from hunger” (MMC_3A). “We had to carry the children who were falling into the water” (Jere_2B). “My children were all sick. One of my children was admitted to the hospital… we had to use a wheelbarrow to convey him through the water (Jere_3A).

#### Disruption of maternal and child healthcare services, including immunization

Due to the flooding, women were unable to access maternal healthcare services, including prenatal and postnatal care. Pregnant women were also unable to access SBA at healthcare facilities during childbirth.

The flood prevented access to the labor room for childbirth and pregnancy tests. This delay hindered our ability to get pregnancy tests and our children vaccinated because the hospital was being used as a displacement center” (Jere_1H). “It took a month for the hospital to become functional again…only then did people start receiving help, including immunizations and ANC” (Jere_3B). “As a result of the flood, we were unable to take our children to the hospital for vaccinations…the vaccines were damaged” (Jere_2C). “We were scheduled to get vaccinated, but we couldn’t because the hospital was still flooded (MMC_3F).

### Perceptions and knowledge of the causes of flooding and climate change

#### Perceived human and environmental drivers of flooding

Participants articulated multiple explanations for the causes of flooding, frequently highlighting human-induced environmental degradation and infrastructural failures. These included blocked drainage systems, indiscriminate waste disposal, construction on waterways, and poor adherence to building regulations. Participants demonstrated awareness of how human activities exacerbate flood risk:

A factor contributing to flooding is the clog or blockage of drainage systems by sachet water waste made from low-density polyethylene and empty water bottles. In addition, many people have built homes inside or very close to riverbanks. In this regard, I can assert that the communities have played a role in exacerbating these issues. (MMC_1A).

Some participants also linked flooding to broader climatic changes, particularly prolonged and intensified rainfall patterns:

With regards to climate change, last year saw significantly more rainfall compared to previous years. Unlike in earlier years, where rainfall would typically stop after two to three months, the rainfall from last year continued for almost six months, indicating a notable change in weather patterns (MMC_1G).

Others emphasized structural and regulatory failures as central causes:

The flooding in Maiduguri stems from multiple factors such as inadequate drainage systems and disregard for building regulations where homes are built on waterways. Ultimately, the blockage of waterways by human activity is a primary cause of the flooding. (Jere_3C).

#### Ambiguity between natural and human causes

Despite recognizing human contributions, participants did not express a unanimous view on whether the flooding was primarily man-made or natural. Several participants described the flood as resulting from a combination of both factors, often conflating natural phenomena with divine intervention:

Definitely, human causes contribute to this issue. We often disregard government regulations when they prevent us from building indiscriminately. However, everyone has witnessed the disaster that results from this behavior” (Jere_1B). “The cause of this flood is from God (Jere_2A).

This ambiguity reflects a broader tendency to merge environmental explanations with spiritual interpretations of natural disasters.

#### Religious and moral interpretations of flooding and climate change

Many participants attributed flooding and climate change primarily to divine will, perceiving these events as acts of God or as punishment for moral transgressions. Climate change was frequently framed within a religious and moral discourse, with some participants interpreting it as a consequence of societal wrongdoing or God’s punishment for sins:

People are committing many sins; for instance, they are engaging in actions that anger God. This can contribute to climate change” (Jere_2H). “This flooding is caused by Allah, whether as a test of our faith or as a consequence of our sins” (MMC_2C). “I think it’s a result of our own mischievous acts. God is showing us that if we are to repent, we should do so, and if we don’t repent, we can witness what God is capable of doing (Jere_1A).

These perspectives illustrate how religious beliefs strongly shape local interpretations of environmental change and disaster causation.

#### Sources of information on climate change and flooding

Participants reported receiving most of their information about climate change through traditional mass media, particularly radio and television, which were commonly used for public awareness campaigns and informational messaging:

We receive valuable information about climate change from radio and television, as well as organizations like the National Orientation Agency (NOA) (Jere_3D).

Some participants also noted the growing role of digital technology and smartphones as emerging sources of information:

We obtain information from radio and television; however, in today’s knowledge-driven world, most people have access to smartphones, allowing them to easily acquire information directly from their devices (MMC_2E).

**Fig. 1:**
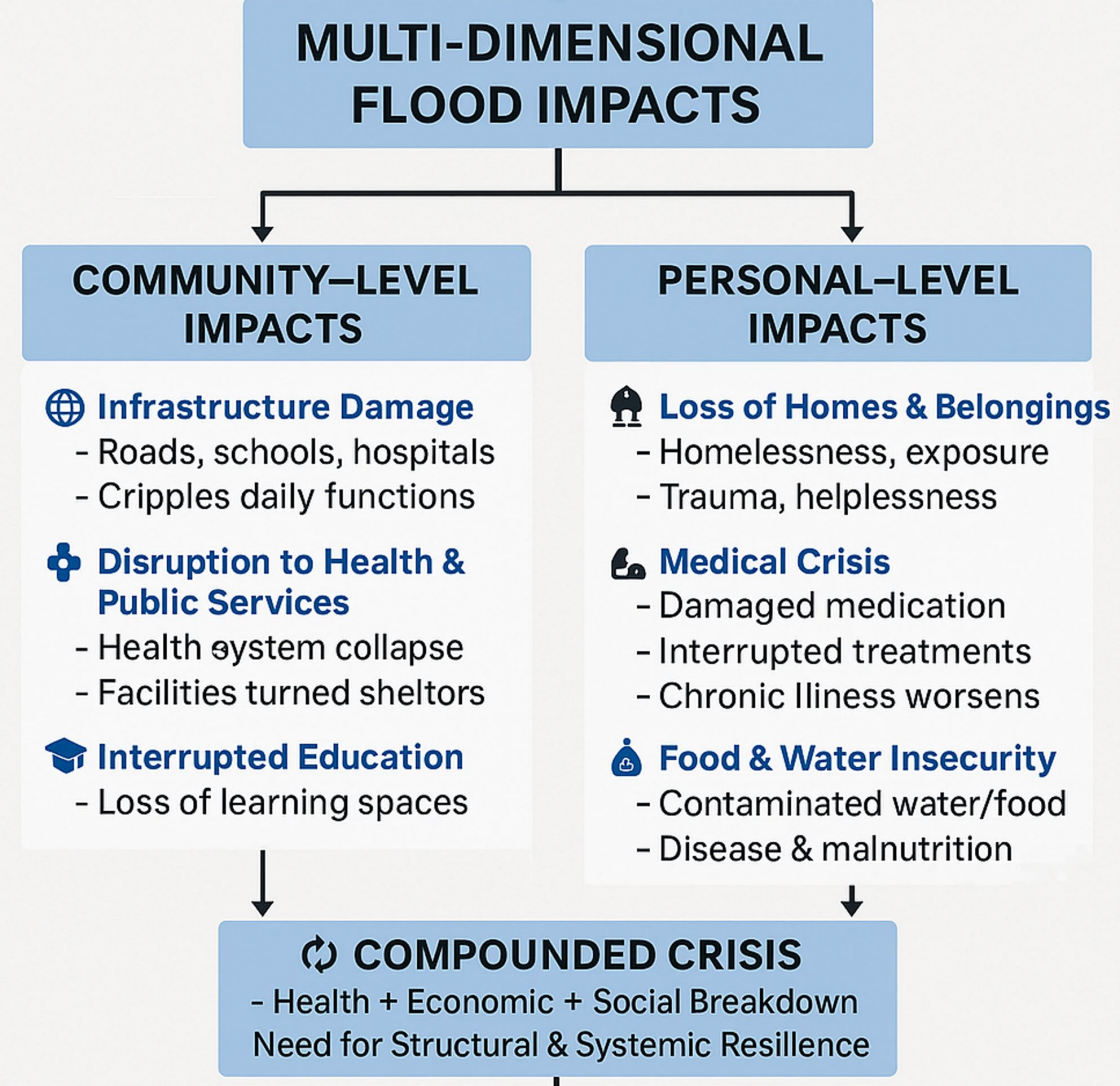
Multidimensional flood impact

As shown in Fig. 1, the combined impacts of personal and community losses on basic survival resources could have devastating consequences for societies grappling with the prolonged armed violent conflict, particularly for vulnerable populations such as children, the elderly, and women.

## Discussion

This qualitative study examined the impact of the 2024 flooding in Borno State on maternal and child healthcare-seeking behavior within a conflict-affected and resource-constrained setting. The findings revealed seven interconnected themes; the disruption to healthcare access and infrastructure; destruction of homes, property and livelihoods; food and water insecurity; immediate and long-term health impacts; disproportionate effects on maternal, child and vulnerable groups; healthcare workforce and supply shortages; and community perceptions of the causes of flooding and climate change. Together, these findings illustrate how the flood triggered a cascading crisis that simultaneously undermined the physical environment, social systems and public health infrastructure, and exposed deep vulnerabilities in service delivery and community resilience.

### Community-level impacts

The findings reflect broader trends observed in climate-related disasters across the Global South. I.e., disruptions in healthcare services, education and infrastructure, mirroring the experiences of societies in other regions where climate shocks have severely impacted public services [27, 28]. The complete shutdown of healthcare delivery demonstrated that limited emergency preparedness and fragile healthcare systems leave communities unable to cope with sudden surges in demand during a crisis. Similar outcomes were observed during the 2022 floods in Pakistan, which damaged over 2,000 healthcare facilities and led to significant outbreaks of disease and disruption to access to medical care [29–31]. Similarly, Cyclone Idai in Mozambique in 2019 caused extensive flooding, destroying several health facilities and leaving communities without essential healthcare services and supplies [32–34].

The destruction of roads, schools and public buildings, which impeded the emergency response and delayed community recovery is consistent with studies from Bangladesh and India, where recurrent monsoon flooding has forced the closure of schools and disrupted the education of millions of children, delaying educational recovery [35–37]. As in other settings, the flood incident revealed that Nigeria´s healthcare and education systems lack the mechanisms and emergency protocols needed to withstand climate stress [38]. Evidence shows that this is not limited to Nigeria, as there is a similar absence of climate-resilient mechanisms in public policies in sub-Saharan Africa and Southeast Asian countries, particularly in conflict-prone or economically marginalized regions [39–41]. Such fragility prolongs recovery and deepens the multidimensional impact of disasters on both physical health and socio-economic stability.

Of note is how the few functioning facilities were repurposed as temporary shelters for displaced community members, resulting in the further displacement of healthcare workers.

A significant devastating outcome was the destruction of medical records, including hospital identification cards (e.g., ANC, immunization records, etc.). This resulted in services being denied where they were available, including childhood vaccinations and maternal care. For example, the University of Maiduguri Teaching Hospital (UMTH), the largest healthcare institution in the state, sustained significant water damage, resulting in the complete suspension of medical activities. Essential medical equipment and infrastructure, including radiotherapy machines, were damaged by the flooding [42, 43]. Similarly, Nakowa Specialist Hospital, one of the largest in the state, was equally and completely submerged, resulting in the loss of laboratory equipment, drugs and patient records [44]. These findings highlight the impact of flooding on healthcare infrastructure and the subsequent disruption to services, particularly in the provision of maternal and child healthcare.

The flooding had a particularly negative impact on maternal and child healthcare, with many pregnant women missing essential ANC services and children missing scheduled immunizations. The flood-induced disruptions meant that pregnant women were at increased risk due to the inaccessibility of services. These disruptions have potentially long-term consequences for public health and demonstrate the impact of damaged records, a lack of personnel and supplies on critical care. Furthermore, around two million doses of vaccine doses were lost when cold chain storage facilities were damaged by the flood, reversing progress in vaccination delivery [43]. Consequently, immunization services declined due to factors such as vaccine stock-outs and a lack of electricity affecting cold chains.

These findings highlight the urgent need for a public health system that can withstand the effects of climate change, including emergency protocols for managing maternal and childcare during disasters. Similarly, community-based support systems must be strengthened to ensure the safety and care of the most vulnerable people during natural disasters.

### Household and personal-level impacts

At the household level, the flood had devastating personal consequences. Many families lost their homes, belongings and livelihoods, which had an immediate impact on their health, nutrition, and psychological well-being. The emotional impact is particularly evident, as participants described how the trauma of destruction affected their health and disrupted their family life. Similar psychosocial and material hardships have been reported in Pakistan, South Africa, Brazil, Niger, Guatemala, and Bolivia [45, 46].

A key finding was the dual impact of disease: exposure to contaminated floodwater, and the destruction of medicines and health facilities. Even facilities that remained structurally intact were often unusable due to contaminated drugs or disrupted supply chains. This exacerbated the vulnerability of populations with chronic illnesses and those requiring continuous maternal or pediatric care. As the findings shows, floodwaters turned basic survival efforts into health hazards, creating a vicious cycle of illness and inadequate care. Similar crises have been reported in Sudan’s Zamzam region, where flooding has inundated sanitation facilities and thereby increased the risk of cholera and other disease outbreaks amidst the already dire conditions created by the ongoing war [47, 48]. Similarly, in eastern Congo, flooding has led to food insecurity and an increased risk of disease due to contaminated water sources [49–52].

The findings reflect the fact that floods can simultaneously act as a crisis with threefold effects on health, the economy and society. The combined effects of infrastructure destruction, service interruption and deterioration of personal health and security underscore the importance of resilient healthcare systems and infrastructure, effective disaster preparedness, and efficient emergency response systems. Most importantly, the experiences of affected populations reveal that recovery encompasses more than rebuilding structures; it also involves restoring the social fabric and systems that sustain communities’ lives and dignity, including healthcare services such as maternal and child healthcare. Nigeria’s healthcare system must integrate climate-resilience mechanisms into the national health agenda. Furthermore, future disaster preparedness must prioritize both structural resilience and the capacity to adapt public health and essential services during crises.

### Vulnerable populations and maternal and child health

Due to its disproportionate impact on pregnant women, children, and the elderly, who faced heightened health risks and barriers to care, it was children who suffered most from widespread illnesses such as fever, diarrhea and malnutrition caused by hunger and consuming contaminated water. This is because they are especially vulnerable due to their dependency on caregivers and weaker immunity systems. Studies of similar flood events have found an excess risk of infant mortality of 5.3 additional deaths per 1,000 births over several decades [53, 54]. These findings emphasize or explain the dangers of flooding and the spread of associated diseases among vulnerable groups.

The Maiduguri floods posed an additional layer of difficulty for pregnant women. Many gave birth enroute to safer locations, without the assistance of SBAs and in unhygienic conditions. The outcome showed that many had experienced miscarriages and stillbirths due to trauma, infection, malnutrition and even exhaustion. Furthermore, pregnant women were at significant risk to their health due to limited access to pre- and postnatal care and heightened stress levels. These factors contributed to adverse pregnancy outcomes (e.g., low birth weight, preterm birth, stillbirth, etc.) in Maiduguri, especially in disaster situations. Evidence shows that floods increase the risk of low birth weight and gestational hypertension among affected populations [55].

### General health effects

The health consequences extended beyond maternal and child health to the wider population. The flooding increased waterborne diseases, such as typhoid, cholera and malaria, as well as skin conditions (e.g., scabies), joint pain and fevers. This was largely attributed to exposure to contaminated floodwater and unsafe drinking water, as well as an inability to maintain hygiene or access medical treatment. These findings are consistent with evidence linking floods to increased transmission of waterborne and vector-borne diseases [56–59]. The lingering wetness and cold conditions after the flood exacerbating respiratory and rheumatic conditions, particularly among children and the elderly could be attributed to direct contact with polluted water, resulting in dermatological issues, while damp and unsanitary conditions lead to musculoskeletal discomfort [60, 61].

### Communities’ perceptions of religion and climate change

The participants´ interpretations of the causes of flooding revealed a complex interplay between belief, local experience and scientific understanding. Many participants described the flood as an act of God or a divine test, reflecting their deeply held religious beliefs regarding the interpretation of climate-related phenomena. Such interpretations pose a challenge for scientific and climate change communication, as they can influence how communities perceive and respond to environmental change.

Several participants viewed the flood as God’s punishment for human disobedience. This aligns with previous studies showing that both Christian and Muslim communities in Nigeria perceive environmental disasters, including desertification drought and flooding, as manifestations of God´s displeasure at humanity´s neglect of environmental stewardship [62, 63]. These perceptions underscore the importance of incorporating religious perspectives into climate change communication strategies to raise public awareness and encourage collective action in response to climate change-related events.

While a few individuals demonstrated an understanding of climate change, several others could not define it clearly. The interplay between scientific knowledge and moral or religious explanations further complicated this understanding. While some participants correctly identified deforestation, changing rainfall patterns, and increased heat as signs of climate change, many attributed these changes to divine will rather than human activity.

Most participants reported receiving information about climate change through radio and television, particularly in rural and semi-urban areas. However, these media outlets often do not provide in-depth education, leading to mixed levels of understanding. Other studies have reported that religious beliefs, which can distort scientific communication, are widespread, with communities attributing climate events to divine punishment or moral decay [63, 64].

Similar findings have been documented in other Nigerian regions, where communities recognize environmental changes, such as increased heat and erratic rainfall, but struggle to connect these changes to broader climate change processes [65, 66]. In northeastern Nigeria, this knowledge gaps especially in relations to healthcare system can be attributed to low levels of education, limited access to scientific information, social norms, low literacy, trust deficit, and competing traditional or religious narratives [67–76].

Addressing these challenges requires communication that is sensitive to culture, acknowledges religious and moral frameworks, and improve access to scientific knowledge. Bridging together faith-based and scientific perspectives could strengthen public understanding and foster communities to adapt more effectively to climate-related disasters.

Overall, the findings of this study have important implications for policy and public health, particularly with regard to strengthening climate-resilient health systems in conflict-affected areas. There is an urgent need to integrate disaster preparedness and climate adaptation into national healthcare policies, with a particular focus on protecting maternal and child healthcare services during emergencies. This requires investment in resilient healthcare infrastructure, safeguarding of medical supply chains (especially the cold chain systems) and digitization of healthcare records, to prevent service disruption. In addition, strengthening primary healthcare and community-based systems, such as training community healthcare workers and establishing mobile or emergency care units, can ensure continuity of care when facilities are inaccessible. Public health strategies should prioritise culturally and religiously sensitive risk communication, leveraging the influence of trusted community and faith leaders to improve understanding of climate change and promote adaptive behaviors. Furthermore, a multisectoral approach that integrates healthcare, water, sanitation and social protection systems is essential to mitigate the cascading impacts of floods, reduce disease outbreaks and enhance the resilience of vulnerable communities.

The study has its limitations. The findings were derived from communities affected by the 2024 flooding only and may not represent the views of other regions in Borno State or Nigeria as a whole. Participant recruitment depended on local security conditions, which may have excluded more remote and/or severely affected areas. As with all qualitative research, the findings are context-specific and reflect the participants’ perspectives. When interpreting how these findings apply to the present-day situation, the temporal gap between data collection and the current state of climatic conditions and conflict should be considered, given the evolving nature of the study setting. However, this gap does not diminish the study’s value. Furthermore, methodological rigour was ensured by triangulating participant categories and achieving thematic saturation. Ethical considerations were maintained throughout to preserve the authenticity of the participants’ voices.

Future research should prioritize investigating the coping mechanisms and adaptive strategies used by households and communities during and after climate-related disasters, especially in conflict-affected settings. Such studies could examine how individuals navigate disrupted healthcare systems, such as using informal care networks, traditional medicine, community support structures and alternative healthcare pathways. Understanding these strategies would provide valuable insights into local resilience, highlight areas where support systems are lacking, and inform the design of context-specific, community-driven interventions that strengthen preparedness, response, and recovery in similar high-risk environments.

## Conclusion

The Maiduguri floods of 2024 exposed a deeply interconnected crisis, revealing how vulnerable communities experience cascading failures when climate disasters strike regions that are inadequately prepared, particularly those affected by conflict. The destruction of infrastructure and disruption to essential services, such as healthcare, education and transportation, has crippled public systems and intensified human suffering. These findings reflect a broader pattern observed across the Global South, where non-climate-resilient infrastructure and weak institutional systems are unable to support communities during climate change related crises.

The impact of the flood extended beyond physical destruction to encompass psychological trauma and the collapse of critical healthcare access for vulnerable groups, especially children and pregnant women, as well as posing threats to basic survival. Contaminated food, water and medical supplies created life-threatening conditions for those already most at-risk, including those with chronic illnesses. These experiences underscore the urgent need for an integrated approach to climate adaptation, strengthening the healthcare system, and disaster preparedness that protects both infrastructure and essential life-saving services.

The crisis was compounded by delayed institutional responses, deep structural vulnerabilities, and cultural interpretations of the disaster that will influence future preparedness and mitigation efforts. Environmental mismanagement, inadequate infrastructure and poor disaster response coordination contributed to long-term socioeconomic and health impacts. The 2024 Maiduguri floods highlight the necessity of investing in climate-resilient healthcare systems, community-centered recovery and disaster response, and culturally sensitive communication strategies, in order to strengthen sustainable resilience in the face of future climate disasters.

## Supplementary Information

Below is the link to the electronic supplementary material.

Supplementary Material 1

## Data Availability

The datasets used and/or analyzed during the current study are available at [https://osf.io/zs59a/] (https://osf.io/zs59a/).
