# Supplementary material for: Health-seeking amid conflict and climate stress: how climate disaster shapes maternal and child healthcare behavior in fragile and conflict-affected settings
Source: BMC Health Serv Res. 2026 May 20;26:714. doi: 10.1186/s12913-026-14744-3 (PMC13192191; doi:10.1186/s12913-026-14744-3)
Supplement: Supplementary file 1 — Supplementary Material 1 [file 12913_2026_14744_MOESM1_ESM.pdf]

FGD GUIDE

The Influence of Flooding on Healthcare-seeking Behaviour for Maternal and Child Health in Borno State, Nigeria.

Introduction:

A. **DEMOGRAPHICS (Complete individually attendance list table: age, gender, occupation, local of residence, nr children, etc.)**
B. **Moderator Introduction**: Briefly introduce yourself and the team.
C. **Purpose**: Explain the purpose of the discussion—to understand the impact of recent flooding on participants' lives and health-seeking behavior.
D. **Confidentiality**: Reassure participants about confidentiality and encourage open sharing of experiences. Collect informed consent
E. **Duration**: Outline the expected time for the session (90 minutes maximum).

Opening Questions (Icebreaker):

1. Can you briefly introduce yourself and share how long you have lived in this community?
2. Have you or your family experienced flooding in recent years? If so, what was the most significant impact on your household?

Section 1: DIRECT HEALTH IMPACT OF FLOODS

3. In what ways have floods affected your health and the health of people in this community?
4. Have you or your family members experienced any health issues due to the floods? (e.g., waterborne diseases, injuries, mental health concerns)
5. What are the most common health problems that emerge during and after flooding?
6. Have pregnant women, children, elderly, or individuals with chronic illnesses faced specific health challenges?

Section 2: ACCESS TO HEALTHCARE SERVICES

7. How have floods affected access to healthcare services in your community?
8. Were there challenges in reaching hospitals, clinics, or pharmacies during the floods?
9. Did healthcare facilities in your area experience disruptions (e.g., closure, lack of staff, medication shortages)?
10. Did the floods prevented you from getting medical care (for instance, missing children vaccination?)

Section 3: COPING MECHANISM AND COMMUNITY RESPONSE

11. How did your community respond to the health challenges caused by the floods?
12. What coping strategies have families used to manage health-related problems post-flooding?
13. Have NGOs, government agencies, or other organizations provided assistance? If so, how effective has their support been?

Section 4. FLOODING AS AN EFFECT OF CLIMATE CHANGE

1. How will you describe the causes of the flooding in Maiduguri?
    a. Probe: Beliefs about the origins of flooding and whether participants attribute it to climate change.
    b. Probe: Do you think it's a natural occurrence, man-made, or a combination of both?
2. What sources of information do you rely on to learn about climate change and its impacts?
    a. Probe: Identify the channels through which participants receive information, such as media, community programs, or educational institutions.
3. Can you describe any specific experiences or observations you have had regarding any of the effects in your area that you believe are linked to climate change?
    a. Probe: Encourage participants to share personal stories or events that illustrate their understanding of climate change effects.
4. What do you understand by the term "**climate change,**" and how do you think it affects You and your community?
    a. Probe: How will you describe the effects…. give specific examples?

Section 5: Long-Term Effects and Recommendations

14. What are the long-term health consequences of recurrent flooding in this area?
15. What measures do you think should be taken to improve health resilience in flood-prone communities?
16. What support do you believe is needed from the government and health organizations to mitigate health impacts in the future?

Wrapping up

- Is there anything else that you would like to tell me about the recent floods and healthcare services?

Closing:

- Thank participants for their insights and time.
- Reiterate the importance of their experiences in shaping policies and interventions.
- Provide contact information for any follow-up inquiries or support service.
